# Cauda Equina Syndrome as the Initial Presentation of Concurrent Plasmacytoma and Multiple Myeloma

**DOI:** 10.7759/cureus.12888

**Published:** 2021-01-24

**Authors:** Alexandra Pisklakova, Christian Almanzar, Jan - Paul Sambataro, Omar Ansari, Faiza Manji

**Affiliations:** 1 Internal Medicine, Brandon Regional Hospital, Tampa, USA

**Keywords:** multiple myeloma, plasmacytoma, back pain, cauda equina syndrome, spinal cord injury

## Abstract

Multiple myeloma is a hematological malignancy characterized by an abnormal proliferation of monoclonal plasma cells. In some occurrences, plasma cell proliferation results in a solitary lesion (solitary bone plasmacytoma or extramedullary plasmacytoma with minimal bone marrow involvement). Approximately 50% of patients with solitary plasmacytoma develop multiple myeloma within 10 years after the initial diagnosis. While back pain and compression fractures are commonly described presentations of multiple myeloma and plasmacytoma, cauda equina syndrome related to plasma cell infiltration is rare and clinical guidelines are limited. Herein, we present a rare case of a woman with acute cauda equina syndrome (CES) secondary to solitary bone plasmacytoma and multiple myeloma.

## Introduction

Multiple myeloma (MM) is a hematological malignancy characterized by the abnormal proliferation of monoclonal plasma cells. It accounts for about 10% of all the hematological neoplasms and is fairly well-described [1]. In some cases, plasma cell proliferation results in a solitary lesion (solitary bone plasmacytoma (SBP) or extramedullary plasmacytoma (EMP) with no evidence of systemic invasion and minimal bone marrow involvement). 

The International Myeloma Working Group (IMWG) diagnostic criteria for MM requires ≥ 10% clonal bone marrow plasma cells or a biopsy-proven plasmacytoma, plus evidence of one or more multiple myeloma defining events, namely, CRAB (hypercalcemia, renal failure, anemia, or lytic bone lesions) [2]. Given the low incidence of SBP, the diagnostic criteria have been difficult to establish. The current guidelines provided by the IMWG define SBP as biopsy-confirmed plasma cell proliferation in the bone or soft tissue in the presence of normal bone marrow and a normal skeletal survey (except for the primary solitary lesion) [2]. Patients presenting with SBP, especially those cases with minimal bone marrow (BM) plasmacytosis, have a higher risk of developing symptomatic MM. Approximately 50% of patients with SBP and 30% of patients with EMP develop MM within 10 years after the initial diagnosis [3-4]. While back pain and compression fractures are commonly described presentations of MM and SBP, cauda equina syndrome, as a result of plasma cell infiltration, is rare and clinical guidelines are limited [5]. Herein, we discuss the case of a woman who presented with acute cauda equina syndrome (CES) with a subsequent diagnosis of SBP and MM. 

## Case presentation

A 71-year-old female with a past medical history significant for hypertension and gastroesophageal reflux disease (GERD) presented to the Emergency Department (ED) with a six-week history of progressive back pain. Her symptoms were associated with constipation, ataxia, saddle anesthesia, as well as bowel and bladder incontinence. Prior to her admission, the patient had been evaluated by a primary care physician who recommended stool softeners without addressing the more alarming symptoms.

**Figure 1:**
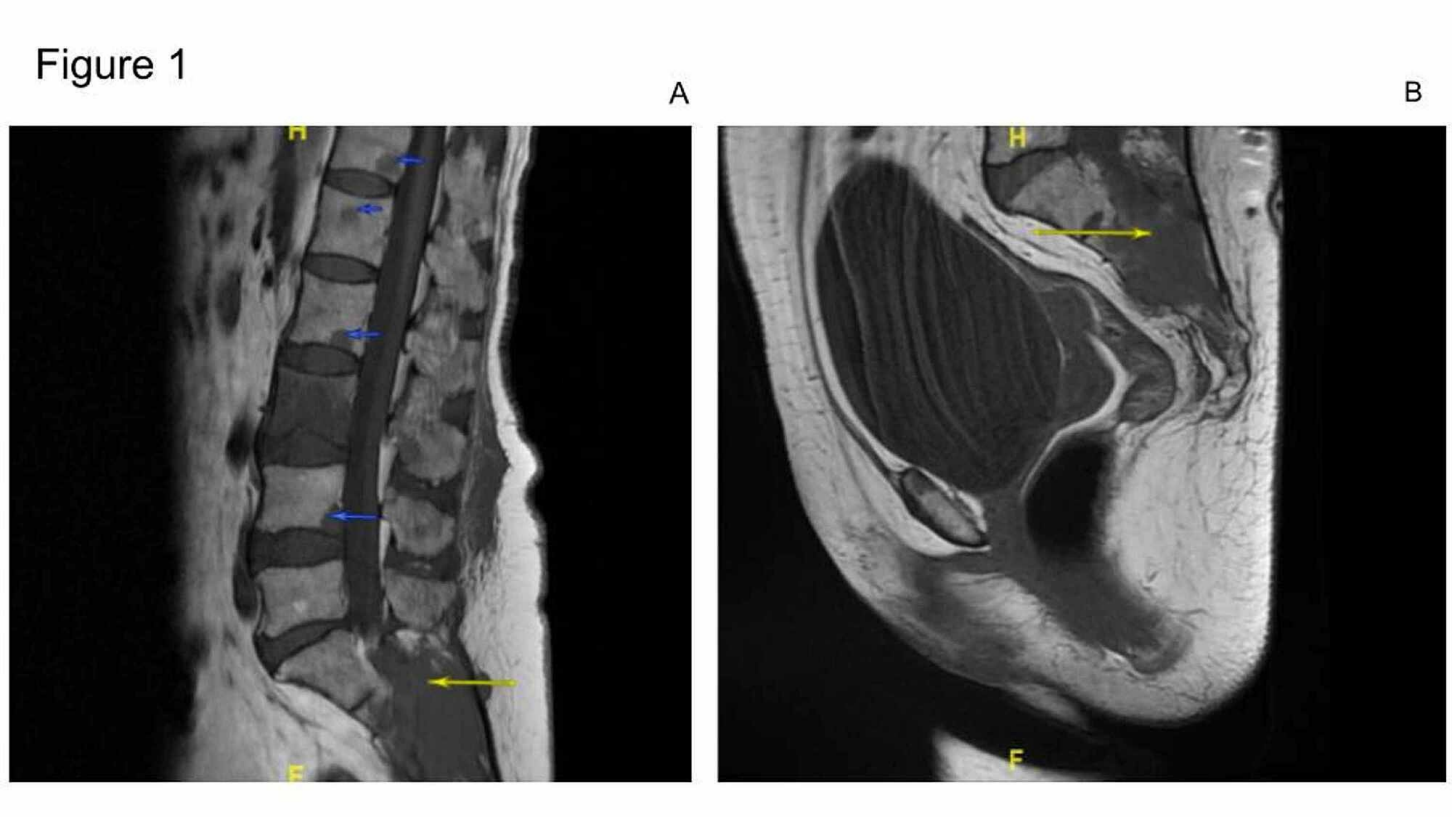
Contrast-enhanced magnetic resonance imaging (MRI) of the lumbar spine and sacrum Contrast-enhanced sagittal MRI images showing innumerable destructive lesions throughout the lumbar spine and sacrum (yellow arrows, Figure 1A) with a 5.6 x 3.5 cm destructive lesion involving the posterior S1, S2, and S3 segments of the sacrum with extension into the sacral spinal canal (blue arrows, Figure 1A-B).

Upon physical examination, the strength in bilateral upper and lower extremities was noted to be 4/5. Patellar and Achilles deep tendon reflexes were 1+ bilaterally. Sensory examination was significant for a decreased light touch, pinprick, position, and temperature in the distribution of the S1 - S3 dermatomes. On the basis of these findings, magnetic resonance imaging (MRI) of the lumbar spine was performed in the ED (Figure 1). On MRI, numerous destructive lesions throughout the lumbar spine and sacrum were observed (Figure 1A). Of particular significance was a 5.6 x 3.5 cm destructive lesion involving the posterior S1, S2, and S3 segments of the sacrum with extension into the sacral spinal canal (Figure 1A-B).

Computed tomography (CT) of the abdomen, pelvis, and chest showed only osseous metastasis localized to the lower thoracic and lumbar spine, sacrum, and iliac bones. Surprisingly, laboratory studies were pristine with only a slight leukocytosis of 13,000 cells/m^3^ and a mildly elevated calcium of 10.3 mg/dL (11.4 mg/dL corrected) with normal creatinine and hemoglobin levels. 

The patient underwent a percutaneous sacral mass biopsy and eventual sacral laminectomy for epidural tumor resection and thecal sac decompression. There was a large amount of tumor infiltration in the lateral sacral lamina, as well as the paraspinous musculature. The thecal decompression was carried out inferiorly through S3 under the guidance of fluoroscopy. Complete resection was performed with no immediate complications.

**Figure 2:**
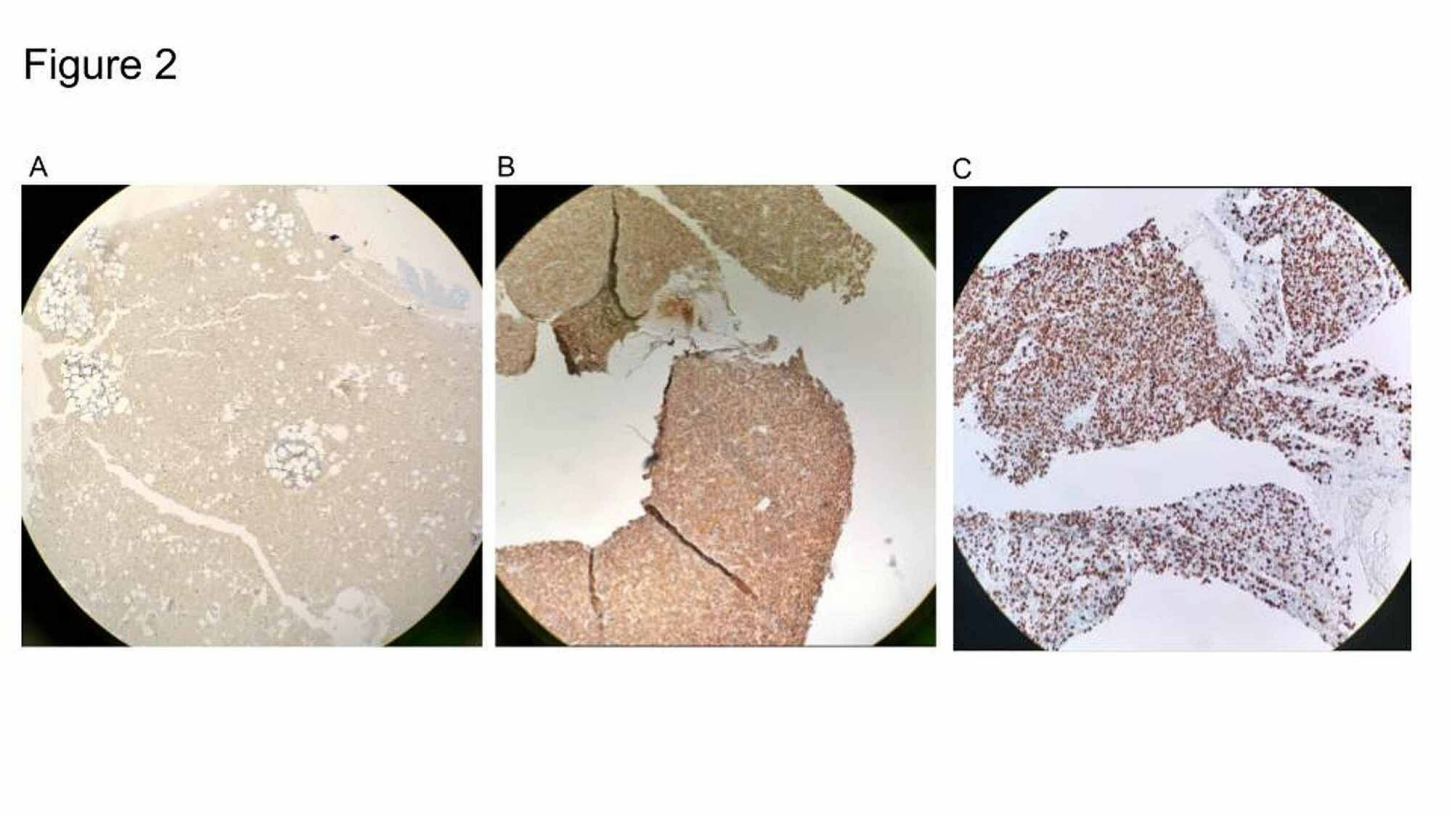
Immunohistochemistry staining of bone marrow and sacral mass biopsy (A) Immunohistochemistry staining of bone marrow for CD138 revealed approximately 30% of plasma cells with interstitial distribution and formed a small compact sheet; (B) immunohistochemistry staining of the sacral mass biopsy showed compact sheets of atypical plasma cell proliferation diffusely positive for CD138 and (C) CD56 with more than 80% of Ki-67-positive cells.

Pathology review confirmed plasmacytoma: positive sheets of atypical plasma cells with immunohistochemistry staining diffusely positive for CD138 and CD56 with increased Ki-67 positive cells (over 80%) (Figure 2B-C). Staining for CD20 and CD3 highlighted a small number of background lymphocytes. A bone marrow biopsy demonstrated atypical plasmacytosis. Flow cytometry was remarkable for an abnormal clonal plasma cell population of CD38++, CD138+, CD19-, and CD56+. Immunostaining was positive for CD138 with approximately 30% of plasma cells immunoglobulin A (IgA) kappa with normal karyotype on cytogenetics (Figure 2A).

Fluorescence in situ hybridization (FISH) showed immunoglobulin heavy chain (IgH) translocation with abnormal fibroblast growth factor receptor-3 (FGFR3), a gain of 1q21, and monosomy for chromosome 13. 

Treatment with lenalidomide/bortezomib/dexamethasone (RVD-lite) and monthly Zometa® (zoledronic acid) was initiated, given the patient's performance status and advanced age. Bowel and bladder incontinence continued to persist despite decompression surgery, leading to further evaluation by radiation oncology for possible radiation treatment. Bone marrow transplantation was discussed with the patient as well. However, she opted to complete radiation therapy and several courses of RVD-lite first. At this time, the patient has tolerated chemotherapy well with minimal side effects and plans to start radiation treatments.

## Discussion

This case highlights MM and SPB as a potential cause of acutely deteriorating neurologic function in patients with lower back pain (LBP), especially in a primary care office setting. Early recognition of compression symptoms and identification of the underlying cause is crucial, as delay in treatment can be devastating. MRI is the first line of investigation in a patient with red flag symptoms, such as those associated with spinal cord compression and cauda equina syndrome - lower back pain, bilateral sciatica, saddle sensory disturbances, bladder and bowel dysfunction, and loss of sensory and motor function in the lower extremities. The prevalence of these serious spinal pathologies has been estimated to be around 5% of newly diagnosed MM patients [6-8].

MM, SBP, and EMP result from the uncontrolled proliferation of plasma cells. However, they are not homogeneous and the approach to management is quite varied and not well-defined. MM and SBP may both present with axial skeletal lesions and with progression into MM occurring in the majority of the patient population. In view of the rarity of this disease, definitive treatment guidelines have not yet been established. Currently, radiation therapy (RT) and surgery are the first-line treatments of SBP [9]. Emergency surgical intervention, such as decompressive laminectomy, is considered to be the first line of treatment in the event of resultant neurological compromise due to spinal cord compression or CES. The treatment modalities implemented in our case have been documented in the medical literature [5, 10]. RT is a preferred treatment modality for patients who are not considered to be surgical candidates, as well as an adjuvant treatment following surgical decompression. The effectiveness of adjuvant chemotherapy is controversial and is used for refractory disease, as well as for the treatment of SBP that has progressed into MM. Recurrent solitary plasmacytoma at different sites may be treated with additional RT, but patients with more extensive disease or early relapse may benefit from systemic therapy and/or autologous stem cell transplant (ASCT), as indicated for myeloma [11]. 

## Conclusions

SBP is an exceedingly rare plasma cell neoplasm with an annual incidence of hundreds of new cases. Clinical presentation is often typified by pathologic fractures and typical laboratory findings. As osteoblastic lesions are commonly found in the spine, lower back pain is not an unusual presentation. On the other hand, cauda equina syndrome in MM and SBP is an uncommon and potentially devastating presentation resulting from compression of the cauda equina in the spinal canal. It can be prevented by early diagnosis, which requires a high index of suspicion on the part of clinicians, including primary care providers. In this report, we present a case with the complex nature of cauda equina syndrome resulting in permanent bowel, bladder, and neurological dysfunction. This case emphasizes the need for a thorough investigation in the presence of the triad: back pain, saddle anesthesia, and urinary incontinence.
